# Imaging the where and when of tic generation and resting state networks in adult Tourette patients

**DOI:** 10.3389/fnhum.2014.00362

**Published:** 2014-05-28

**Authors:** Irene Neuner, Cornelius J. Werner, Jorge Arrubla, Tony Stöcker, Corinna Ehlen, Hans P. Wegener, Frank Schneider, N. Jon Shah

**Affiliations:** ^1^Institute of Neuroscience and Medicine - 4, Forschungszentrum Jülich GmbHJülich, Germany; ^2^Department of Psychiatry, Psychotherapy and Psychosomatics, RWTH Aachen UniversityAachen, Germany; ^3^JARA – Translational Brain MedicineAachen, Germany; ^4^Department of Neurology, RWTH Aachen UniversityAachen, Germany

**Keywords:** resting state networks, Tourette, tics, basal ganglia, cortico-striato-thalamo-cortical circuit

## Abstract

**Introduction:** Tourette syndrome (TS) is a neuropsychiatric disorder with the core phenomenon of tics, whose origin and temporal pattern are unclear. We investigated the When and Where of tic generation and resting state networks (RSNs) via functional magnetic resonance imaging (fMRI).

**Methods:** Tic-related activity and the underlying RSNs in adult TS were studied within one fMRI session. Participants were instructed to lie in the scanner and to let tics occur freely. Tic onset times, as determined by video-observance were used as regressors and added to preceding time-bins of 1 s duration each to detect prior activation. RSN were identified by independent component analysis (ICA) and correlated to disease severity by the means of dual regression.

**Results:** Two seconds before a tic, the supplementary motor area (SMA), ventral primary motor cortex, primary sensorimotor cortex and parietal operculum exhibited activation; 1 s before a tic, the anterior cingulate, putamen, insula, amygdala, cerebellum and the extrastriatal-visual cortex exhibited activation; with tic-onset, the thalamus, central operculum, primary motor and somatosensory cortices exhibited activation. Analysis of resting state data resulted in 21 components including the so-called default-mode network. Network strength in those regions in SMA of two premotor ICA maps that were also active prior to tic occurrence, correlated significantly with disease severity according to the Yale Global Tic Severity Scale (YGTTS) scores.

**Discussion:** We demonstrate that the temporal pattern of tic generation follows the cortico-striato-thalamo-cortical circuit, and that cortical structures precede subcortical activation. The analysis of spontaneous fluctuations highlights the role of cortical premotor structures. Our study corroborates the notion of TS as a network disorder in which abnormal RSN activity might contribute to the generation of tics in SMA.

## Introduction

Tourette syndrome (TS) is a neuropsychiatric disorder characterized by multiple motor and one or more vocal/phonic tics (Singer, 2005; Neuner and Ludolph, 2011; Robertson, 2012). Tic onset occurs during childhood, typically between the ages of 4–6 years. In up to 60–70% of the patients the tics subside in adulthood, leaving only a small percentage of cases developing chronic adult TS. The severity of tics waxes and wanes over time. Patients often report that stress and teasing by others worsen tics, whereas focused activities such as reading or physical exercise reduce their occurrence. TS is often accompanied by comorbidities such as obsessive-compulsive disorder (OCD), depression and attention-deficit-hyperactivity disorder (ADHD) (Khalifa and von Knorring, 2003; Robertson, 2012). However, tics respond well to treatment with typical and atypical neuroleptics (Kawohl et al., 2009a,b; Roessner et al., 2011; Neuner et al., 2012; Robertson, 2012), and in adult cases resistant to pharmacotherapy, deep brain stimulation shows promising results (Vandewalle et al., 1999; Neuner et al., 2009; Ackermans et al., 2011; Müller-Vahl et al., 2011; Cannon et al., 2012; Ackermans et al., 2013a,b).

In the pathophysiology of TS, the cortico-striato-thalamo-cortical circuit plays an important role (Leckman, 2002; Singer, 2005; Cavanna and Termine, 2012). The neuroanatomy of tics in TS has received particular attention in imaging studies, highlighting a network of frontal areas, basal ganglia, insula and cerebellum. Such findings are compatible with the notion that TS is the result of a failure in network maturation, particularly of the fronto-striatal-thalamic-cortical loop. In TS, structure and function seem to be tightly related: on the structural level, a relative gray matter reduction in orbitofrontal, anterior cingulate and ventrolateral pre-frontal cortices bilaterally in adult TS patients has been observed (Draganski et al., 2010), along with cortical thinning of the limbic mesial temporal lobe (Worbe et al., 2010). Structural diffusion tensor imaging (DTI) studies report an involvement of the corticospinal tract, and of the underlying white matter under the supplementary motor area (SMA), the pre- and post-central gyrus and the ventral-post-erolateral nucleus of the right thalamus (Thomalla et al., 2009; Neuner et al., 2010b). Hypertrophy of the limbic and pre-frontal cortices, and a smaller corpus callosum are associated with less pronounced tics in children with TS (Plessen et al., 2009). Regarding the function, amygdala hypersensitivity and alterations of its structure and functional connectivity have been described (Neuner et al., 2010a; Werner et al., 2010).

A recent functional magnetic resonance imaging (fMRI) study on tics concluded that “tics are caused by the combined effects of excessive activity in motor pathways (including the sensorimotor cortex, putamen, pallidum, and substantia nigra) and reduced activation in control portions [caudate nucleus and anterior cingulate cortex (ACC)] of cortico-striato-thalamo-cortical circuits” (Wang et al., 2011). The role of the ACC in tic suppression was highlighted in a single case fMRI study (Kawohl et al., 2009c). Furthermore, Bohlhalter and colleagues described that 2 s prior to a tic an activation pattern consisting of the insular cortex, SMA and parietal operculum is observed; at tic onset, a neuronal network formed by sensorimotor areas including the superior parietal lobule bilaterally and the cerebellum was activated (Bohlhalter et al., 2006). An early video-controlled ^15^O-PET (positron emission tomography) study by Stern and colleagues identified the following neuronal network active with the occurrence of motor tics: medial and lateral premotor cortices, ACC, dorsolateral-rostral pre-frontal cortex, inferior parietal cortex, putamen and nucleus caudate, as well as primary motor cortex, sensorimotor cortex, superior temporal gyrus, insula, and claustrum (Stern et al., 2000). These findings were later replicated by Lerner et al. who also reported insula activation (Lerner et al., 2007). Using ^18^FDG-PET, Braun and co-workers described decreased metabolic rates in the orbitofrontal, inferior insular and parahippocampal regions in TS patients (Braun et al., 1993). Further metabolic decreases were observed in the nucleus accumbens and the ventromedial caudate nucleus. However, an increased metabolic activity was found in the supplementary motor, lateral premotor and Rolandic cortices.

The lack of inhibition has been proposed as the main underlying principle in TS—affecting impulses, movements, thoughts, attention, and behavior (Cohen and Leckman, 1991). This hypothesis was supported in a study by Church and colleagues, in which immature patterns of connectivity were described in adolescent TS patients, particularly in the frontoparietal network, which is thought to maintain adaptive online control. In the same study aberrant connections were found in regions belonging to the frontoparietal network, possibly resulting in deficient inhibition that might result in tics (Church et al., 2009).

Although all the previously mentioned neuroimaging studies (Supplementary table 1) delineate neuronal networks as the possible underlying cause of tics, the origin and temporospatial development before, during and after a tic remain unclear.

Another factor that remains unknown in TS is how resting state networks (RSNs) influence the generation of tics, particularly, whether they contribute to the postulated motor hyperexcitability or the failure of inhibition. The concept of RSNs has gained momentum over the past years (Biswal et al., 1995; Raichle et al., 2001; Greicius et al., 2003; Damoiseaux et al., 2006; Buckner et al., 2008; Damoiseaux and Greicius, 2009; Rosazza and Minati, 2011). They are defined as coherent and spontaneous fluctuations of the human brain at rest. These RSNs have been identified by means of several imaging methods, particularly fMRI, where strong correlations can be identified among blood-oxygen-level-dependent (BOLD) signal fluctuations of distinct regions of the brain in the resting state, as well as in PET studies (for review Rosazza and Minati, 2011). Abnormalities of RSNs have been related to neurological (Boly et al., 2009; Bonavita et al., 2011; Werner et al., 2014) and psychiatric diseases (Bluhm et al., 2007; Pearlson and Calhoun, 2009). Among the different RSNs that have been described the default-mode network (DMN) has a salient role (Raichle et al., 2001; Greicius et al., 2003; Buckner et al., 2008; Damoiseaux and Greicius, 2009; Rosazza and Minati, 2011). It was largely demonstrated that this network is more intensely active under resting conditions and relatively de-activated whenever the participant was involved in any active task. The functional integrity of the DMN has been reported to be altered in the presence of movement disorders such as Parkinson's disease (Tessitore et al., 2012; Esposito et al., 2013) and Huntington's disease (Wolf et al., 2012; Werner et al., 2014). The actual status of the RSNs has demonstrated to influence motor symptoms in patients suffering from Parkinson's disease (Esposito et al., 2013). The association between altered RSNs in movement disorders raises the question whether alterations in RSN activity also contribute to the occurrence of tics or disease severity in general.

Focussing on tics, and based on the concepts of RSNs and the existing structural and functional imaging data on TS, this study aims to address the following research questions:

1. Where do tics originate and what are the temporal dynamics of tic generation, given that tics can be detected in a video-controlled approach during fMRI?
2. Could the RSNs and particularly the DMN be identified in TS patients in the presence of tics?
3. Does RSN strength in tic-related areas as identified in (1) correlate with clinical markers of TS, such as the Yale Global Tic Severity Scale (YGTSS)?

## Materials and methods

FMRI data from 36 adult TS patients were collected. Functional MRI data were subjected to two different analyses. Firstly, we investigated the origin and neuronal correlates of tic generation using an innovative and objective approach controlled by video. Two MR-compatible cameras inside the MR scanner monitored the body and the face of the patients during the fMRI data acquisition. The MR compatible video camera system is described in more detail elsewhere (Neuner et al., 2007). For the analysis a standard GLM approach was used including the tic onsets as well as two arbitrarily set preceding time-points (2 and 1 s prior to tic occurrence) as regressors of interest (see below). The time points were set at time intervals as reported for movement disorders in existing literature (Cunnington et al., 2003; Bohlhalter et al., 2006).

Secondly, we aimed to determine the influence of RSN activity on the areas involved in tic-generation as defined above. Here, we used a combination of Independent Component Analysis (ICA) and Dual Regression as described previously (Filippini et al., 2009; Zuo et al., 2010), with disease severity as regressor of interest.

The study was conducted according to the Declaration of Helsinki and under granted approval from the ethics committee of the medical faculty RWTH Aachen, Germany.

### Patient sample “GLM”

Out of the entire cohort of patients, data sets from 10 adult patients (8 males, 2 females; aged mean 32 ± 12.1 (*SD*) years) suffering from moderate to severe TS (according to ICD-10 and DSM-IV criteria, mean YGTSS = 51.5, *SD* = 23.1) were used in the analysis. Data of the other patients had to be excluded because of excessive motion artifacts (movement >3 mm from one volume to the next), deficient video quality (*n* = 2, displaced mirror in one case and insufficient lighting in the other) or low number of tics during data acquisition. For sufficient statistical power, we estimated that a minimum of 20 tic events was necessary. Thus, patients with less than that number of tics were excluded from the analysis. The patients included in this part of the analysis had tics in the range of 23–71 (mean 39.5) during the scanning session.

Full medical and demographic data are presented in Table 1. Eight out of the 10 adult TS patients were medicated. As medication was very heterogeneous with respect to substance classes, we did not include medication as a regressor in our analysis. Clinical evaluation, and inclusion and exclusion criteria were the same as in the (larger) Tourette sample included in the resting state analysis, as described below.

**Table 1:** **Demographic and clinical data of Tourette patients (*n* = 16)**.

**Subject**	**Age**	**Gender**	**OCD**	**ADHD**	**YGTSS total**	**YGTSS motor**	**YGTSS vocal**	**Current daily medication**	**Tic generation analysis**	**Resting state analysis**
1	41	F	No	No	33	13	0	None	Yes	Yes
2	32	M	No	No	67	14	13	Trimipramine 100 mg	Yes	Yes
3	26	M	No	No	40	11	9	Escitalopram 10 mg	Yes	Yes
4	56	F	Yes	No	63	16	7	Citalopram 40 mg, carbamazepine 400 mg	Yes	Yes
5	39	M	Yes	No	80	14	16	200 AMS, tiapride 50 mg	Yes	Yes
6	49	M	No	No	37	11	6	None	Yes	Yes
7	25	M	Yes	No	66	13	13	Citalopram 20 mg, tiapride 50 mg	Yes	Yes
8	27	M	No	No	44	14	0	Trimipramine 50 mg	Yes	Yes
9	23	F	No	No	27	10	7	Aripiprazole 10 mg, Fluoxetine 20 mg	Yes	Yes
10	19	M	No	No	60	16	14	Ziprasidone 80 mg	Yes	Yes
11	36	M	No	No	68	15	13	None	No	Yes
12	47	F	No	No	46	10	6	None	No	Yes
13	25	M	No	No	2	2	0	None	No	Yes
14	27	M	Yes	Yes	63	12	11	None	No	Yes
15	22	F	No	No	57	15	12	None	No	Yes
16	21	M	No	No	62	10	12	None	No	Yes

OCD, comorbid obsessive compulsive disorder; ADHD, comorbid attention deficit hyperactivity disorder; YGTSS, Yale Global Tic Severity Scale.

Subjects were instructed to keep their eyes closed and to think of nothing in particular while lying in the scanner. They were allowed to let their tics occur freely, i.e., not to undertake any effort to suppress them. Tics were recorded by an MR-compatible whole-body video system using two video cameras covering both face and the entire body, using a temporal resolution of 10 frames per second. Video data were scrutinized under the supervision of a board certified expert (IN, neurology and psychiatry). Tic onset times were rounded to 0.5 s increments.

### Functional data acquisition “GLM”

MR-acquisition of functional data (echo-planar imaging) were performed using the following protocols in a 1.5 T Siemens MR scanner (T2^*^-weighted echo-planar images, *TE* = 60 ms, *TR* = 3.2 ms, 30 slices, 10% gap, FOV = 200 mm, in-plane resolution = 3.125 × 3.125 mm, eyes closed, 12 min). A contiguous session without interruptions encompassing 220 volumes was acquired.

A T1-weighted, 3D gradient-echo pulse sequence (MP-RAGE, magnetization-prepared, rapid acquisition gradient echo) with the following parameters: *TI* = 1.2 s, *TR* = 2.2 ms, *TE* = 3.93 ms, 15°flip angle, FOV = 256 × 256mm^2^, matrix size = 180 × 256, 176 sagittal slices generated anatomical images (resolution 1 mm isotropic) of the brain. TS patients were monitored with an MR-compatible camera-system (Neuner et al., 2007) to monitor tic related whole-body motor activity.

From the structural data, we also derived voxel-wise gray matter nuisance regressors for each subject, which were used in all functional data analyses to control for structural differences on the individuals' voxel level (Oakes et al., 2007). Briefly, after tissue-type segmentation using FAST implemented in FSL (Version 4.1, FMRIB's Software Library, www.fmrib.ox.ac.uk/fsl), gray matter images were normalized into standard space and demeaned across the whole group. This yields a numerical value for every subject and every brain voxel indicating the amount of relative gray matter in relation to the group, which can be entered as regressors of no interest in subsequent analyses of fMRI data, thus correcting for the influence of atrophy on an individual and voxel-specific level.

### Functional data analysis “GLM”

FMRI data processing was carried out using FEAT (fMRI Expert Analysis Tool), part of FSL (Version 5.98, FMRIB's Software Library, www.fmrib.ox.ac.uk/fsl). The following pre-statistics processing steps were applied after discarding the first three volumes to allow for magnetic field saturation: motion correction using MCFLIRT; slice-timing correction using Fourier-space time-series phase-shifting; non-brain removal using BET; spatial smoothing using a Gaussian kernel of 6.0 mm (full width—half maximum); grand-mean intensity normalization of the entire 4D dataset by a single multiplicative factor; and high-pass temporal filtering adapted to the frequency of the tics (Gaussian-weighted least-squares straight line fitting, with sigma = 25 s). Additionally to the standard motion correction performed by FLIRT, an additional step to detect and correct “spiky” motion artifacts was used. Volumes with steep motion gradients from one volume to the next were detected by running FSL Motion Outliers (FMRIB's Software Library, www.fmrib.ox.ac.uk/fsl). It included motion correction, calculation of the metric values for each timepoint, thresholding of the metric values using the 75th percentile + 1.5 times the interquartile range to look for outliers and generation of a confound matrix. Thus, this script creates a set of additional regressors flagging critical volumes inside the GLM as time-points of no interest, thus automatically adjusting the degrees of freedom without corrupting the temporal structure of the data.

Time-series statistical analysis was carried out using FILM and local autocorrelation correction. Regressors coding actual tic onsets (tic) as detected by the video analysis were convolved with a double-gamma basis function imitating the shape of the hemodynamic response function (HRF). Actual tic duration was also coded by the regressor. Further regressors comprised time-bins of 1 s duration each (so-called “stick functions”), starting 1 (t-1) and 2 (t-2) s before tic onsets, respectively, and the six motion correction parameters. Regressors t-1 and t-2 were also convolved with the HRF. The analyses for tic, t-1, and t-2 were carried out in separate GLMs/design matrices, due to the highly collinear nature of these regressors, thus losing some specificity.

The following contrasts were computed for each subject: conditions “Tic > baseline,” “(t-1) > baseline,” and “(t-2) > baseline.” “Baseline” in our case was an implicit baseline, e.g., all volumes where no tic activity was detected. Contrast parameter estimates were carried up to a higher-level analysis. Functional data were co-registered onto their structural high-resolution counterparts and then transformed into standard MNI space using FLIRT. Higher-level statistics were performed using FEAT stages 1 and 2 in a mixed-effects model comprising both fixed- and random-effects. The group mean was calculated for each of the lower-level Z-statistics. Images for the group-level contrasts “t-2,” “t-1,” and “Tic” were thresholded using clusters determined by a voxel *Z* > 2.3 and a cluster significance threshold of *p* < 0.05 (corrected for multiple comparisons according to Gaussian random field theory).

Crucially, we argue that despite having a TR of 3.2 s, we are able to delineate neuronal events that are separated by only a time increment of 1 s due to the oversampling of the HRF over the course of the experiment which results from the natural “jitter” of the tic events (Supplementary Figure 1). This jitter leads to a near-complete sampling of the HRF at each time-point. However, due to constraints of the GLM, we needed to enter each regressor into its own GLM (see above). Therefore, no statistical comparison between time-points could be performed (e.g., more activation at 2 s before tic onset vs. 1 s before tic onset). Only qualitative differences could be demonstrated.

In order to further protect our findings against spurious results, we created a set of entirely random regressors using a random sequence of stick functions created by the true random number generator provided by www.random.org. This resulted in a set of regressors, where stick functions with a length of 1 s (similar to the approach mentioned above) were distributed randomly over the duration of the scanning session. Pre-processing and statistical analyses were performed in the same fashion as the functional data. On the second level, data were thresholded using a cluster significance threshold of *p* < 0.05/voxel *Z* > 2.3 (corrected for multiple comparisons as above) and additionally by an uncorrected *Z* > 3.0.

### Patient sample “RSN”

From our cohort of 36 adult Tourette patients (same population as above), data from 16 adult Tourette patients (11 male, 5 female, aged 19–56 years, mean = 32.2, *SD* = 11.2, YGTSS mean = 50.9, *SD* = 19.7) were included in the final analysis, i.e., the same sample as above plus 6 additional patients; see Table 1 for details. 20 data sets were rejected due to excessive motion (movement >3 mm) or imaging artifacts in some data sets. Eight out of 16 patients were medicated.

### Functional data acquisition “RSN”

FMRI data acquisition was the same as described above, i.e., data were acquired in a 1.5 T Siemens MR scanner (T2*-weighted echo-planar images, *TE* = 60 ms, *TR* = 3.2 ms, 30 slices, 10% gap, FOV = 200 mm, in-plane resolution = 3.125 × 3.125 mm, 12 min). In total, 220 volumes were acquired. The subjects were instructed to keep their eyes closed and to think of nothing in particular while lying in the scanner. They were allowed to let their tics occur freely, i.e., not to undertake any effort to suppress them.

### Functional data analysis “RSN”

Standard pre-statistical processing was performed including brain extraction, motion correction, spatial smoothing (FWHM 6 mm), and high-pass temporal filtering (sigma = 100 s), using the respective tools in FSL (FMRIB, FSL Software Library, www.fmrib.ox.ac.uk/fsl). Functional data were linearly co-registered into standard MNI space by the means of the individuals' structural scans. Pre-processed data were concatenated temporally across subjects and then decomposed into spatially independent maps representative for the whole study sample using probabilistic Principal Component Analysis with automatic dimensionality estimation using MELODIC v. 3.09 (Multivariate Exploratory Linear Decomposition Into Independent Components; Beckmann and Smith, 2004). No *a priori* number of components was entered into the analysis, as there was no previous guiding data.

Estimated component maps were thresholded with a probability of *p* > 0.5 by fitting a mixture model to the histogram of intensity values, i.e., false positives were penalized equally to false negatives. Voxels with a *Z*-score > 6 belonging to the respective maps were plotted on a standard MNI-152 brain. “Meaningful” RSNs (i.e., representing neuronal signal as opposed to physiological and non-physiological noise such as vascular, respiratory and motion artifacts) were identified by matching them visually against a previously published set of data encompassing 20 “canonical” RSNs (Smith et al., 2009; http://fsl.fmrib.ox.ac.uk/analysis/brainmap+rsns). In addition, a Jaccard coefficient was calculated between our data and the “canonical” data set, yielding very similar results (data not presented).

In order to test the hypothesis that disease severity and RSN activity are related in tic-related brain areas (particularly in brain areas active prior to a tic), a spatial intersection of RSNs (thresholded at *z* > 4) was created with activation maps generated by the GLM approach from above using tics as regressors (*p* < 0.05 corrected, see below) at the earliest time point prior to a tic we measured (i.e., t-2 s). Basically, this ROI contained exactly that subset of the anatomically most closely matching RSN which in addition to exhibiting spontaneous “resting” activity showed additional neuronal signal just 2 s prior to a tic. These regions of interest were later used as masks in the dual regression analysis as described below.

Dual regression analysis (Filippini et al., 2009; Zuo et al., 2010) was performed as follows: all ICA maps (including artifactual ones) were spatially regressed against the individual fMRI data sets to identify matrices describing subject- and map-specific time-courses and to simultaneously regress out nuisance signal such as CSF and white matter-signal. These time-courses were used to estimate the respective spatial map for each subject (temporal regression) in terms of voxel-wise z-scores. Based on the previous work by Smith et al. (Smith et al., 2009), we selected those maps which represented most closely known and neuroanatomically meaningful RSNs.

The final analysis aimed at correlating YGTSS scores (motor plus vocal) with RSN connectivity measures as represented by the respective individual z-scores in the ROIs generated above. We hypothesized that clinical YGTSS scores had an influence on RSN signal particularly at those spatial locations that are active just prior to a tic. This analysis was carried out using a GLM matrix and non-parametric permutation testing (5000 permutations) in order to test for statistical significance in each ROI (Nichols and Holmes, 2002). Subjects age as well as voxel-wise gray matter values (correcting for atrophy) were entered as nuisance regressors in all the analyses. As we tested a multitude of voxels across three RSNs, we addressed the issue of multiple testing by controlling the false discovery rate (FDR) at *p* ≤ 0.05 (Benjamini and Yekutieli, 2001). Non-parametric testing was used because it is a very robust method making the least assumptions about underlying data structure.

## Results

### Post-scan debriefing

All subjects reported full compliance with the instructions; no self-reports of having fallen asleep were given. None of the subjects reported having suppressed any of the tics.

### Video analysis

Patients (entire cohort of 16 patients eligible for fMRI analysis) exhibited an average of 31.5 tics during the session (range 3–71), resulting in a cumulative tic-time of 175.5 s (i.e., 25,4% of the entire measuring time) with an average duration of 5.6 s per tic. There was a certain correlation between the number of tics inside the scanner and the YGTSS motor + vocal score, which, however, narrowly failed to reach significance (Pearson's r = 0.42, *Df* = 14, *T* = 1.76, *p* = 0.05051, one-sided testing for positive correlation). The correlation between YGTSS motor + vocal and total time spent with tics was far less pronounced (Pearson's r = 0.31, *Df* = 14, *T* = 1.20, *p* = 0.125, one-sided testing for positive correlation).

### Functional data “GLM”

**Figure 1:**
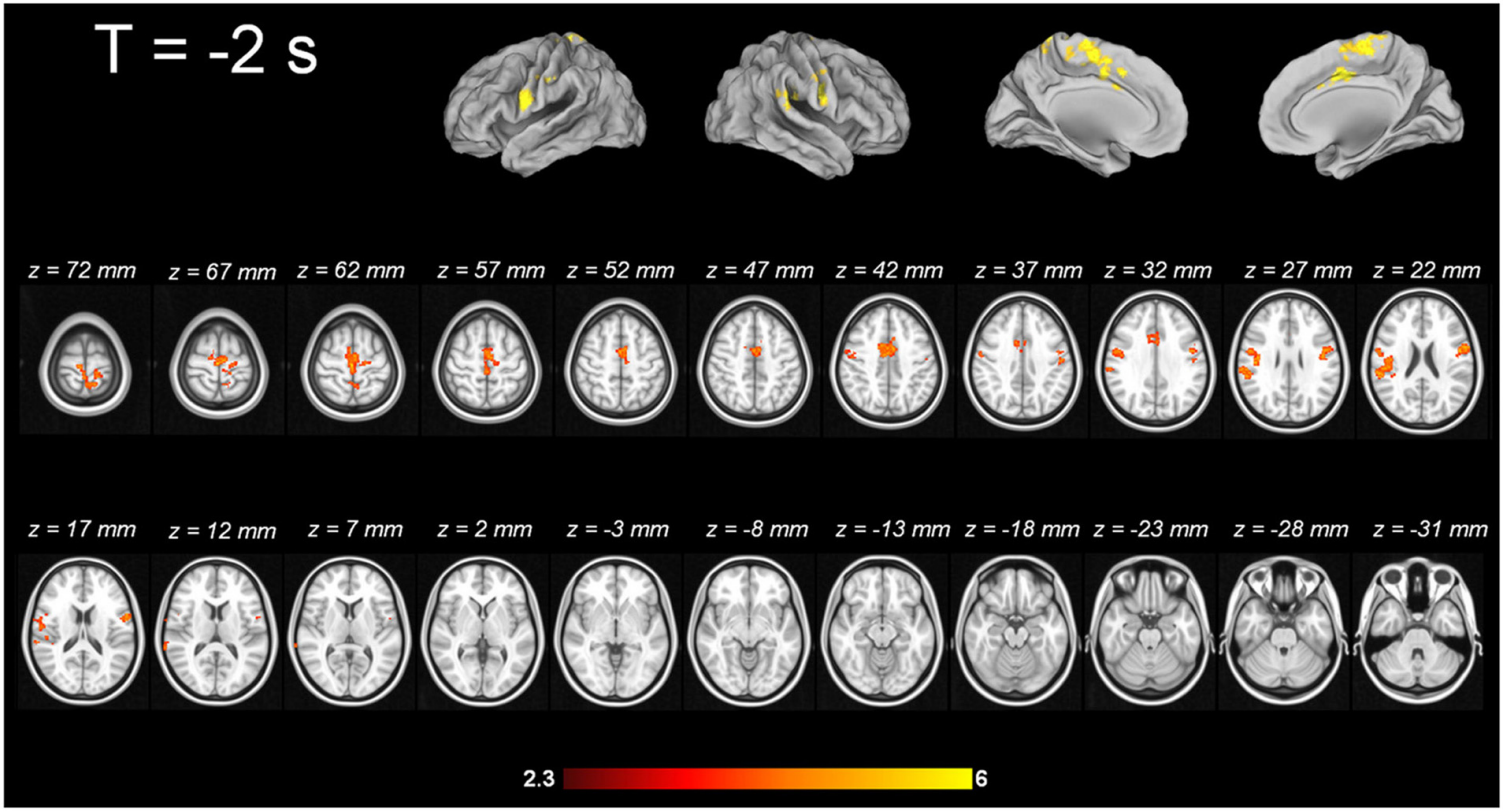
**Tic-related activity 2 s before tic**.

**Figure 2:**
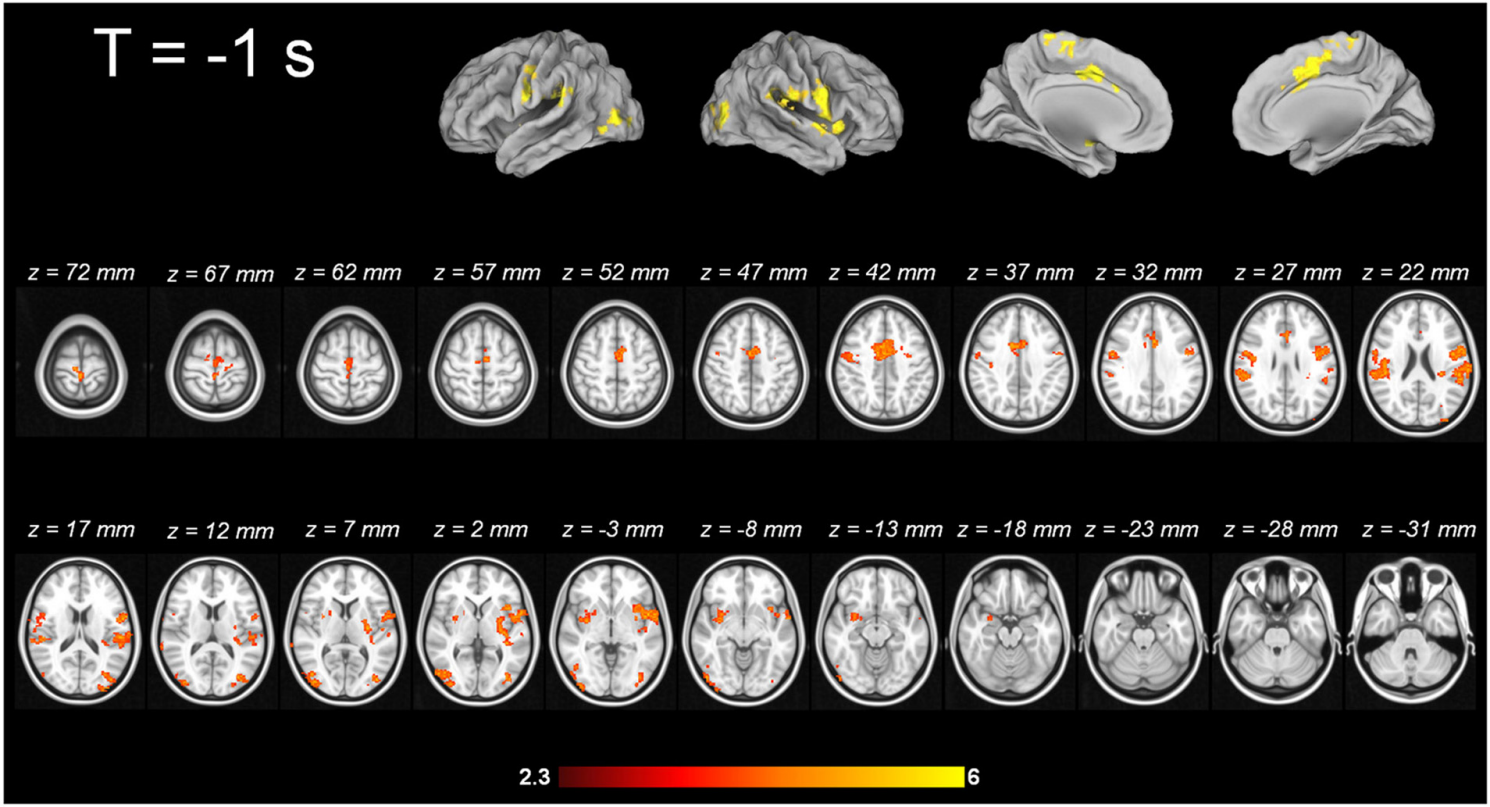
**Tic-related activity 1 s before tic**.

**Figure 3:**
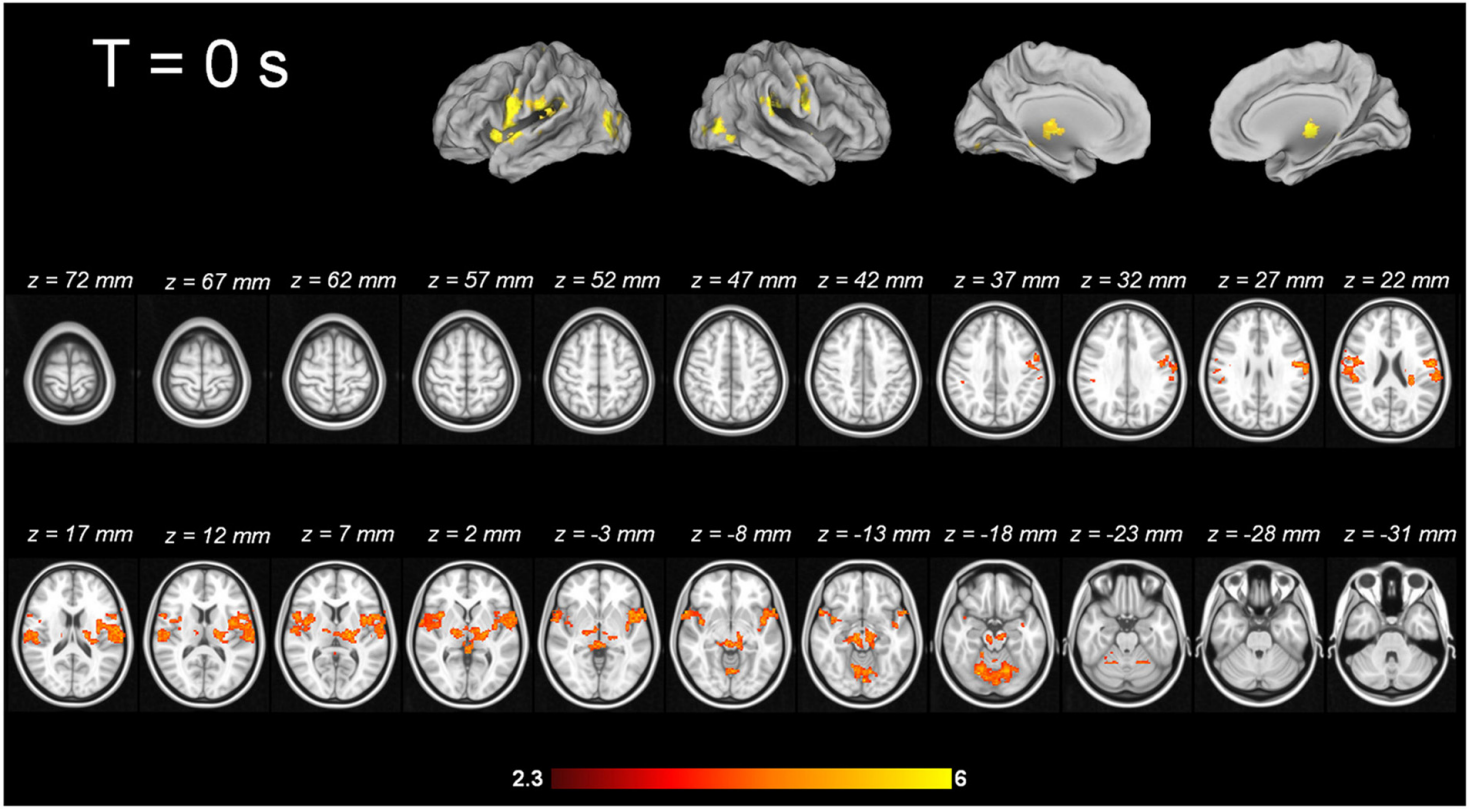
**Tic-related activity**.

For tic-related activity, a spatio-temporally distinct pattern of activation was found (Supplementary Figure 2): 2 s before a tic supplemental motor area (SMA) (BA6 mesial), ventral primary motor cortex (BA6 lateral), primary sensorimotor Cortex (BA3+4) and parietal operculum exhibited activation (Figure 1). One second before a tic, the anterior cingulate, the putamen, the insula, the amygdala, the cerebellum and the extrastriatal-visual cortex exhibited activation (Figure 2). With tic-onset, the thalamus, primary motor and somatosensory cortices and the central operculum exhibited activation (Figure 3). These results are summarized in Table 2.

**Table 2:** **MNI coordinates of clusters with maximal z-scores in the fMRI data analysis of tics in the Tourette sample**.

**Contrast**	**Structures to which each cluster belongs to (Harvard-Oxford Cortical and Subcortical Structural atlases)**	**Number of voxels**	**Max. z-score**	**MNI coordinates**		
				***x***	***y***	***z***
T-2	Left pre-central gyrus	1804	3.73	44	53	70
	Left post-central gyrus					
	Left juxtapositional lobule cortex (formerly left supplementary motor cortex)					
	Left paracingulate gyrus					
	Left cingulate gyrus, anterior division					
	Left precuneous cortex					
	Right pre-central gyrus	1100	3.53	13	50	50
	Right superior temporal gyrus, posterior division					
	Right post-central gyrus					
	Right supramarginal gyrus, anterior division					
	Right supramarginal gyrus, posterior division					
	Right central opercular cortex					
	Right parietal operculum cortex					
	Right planum temporale					
	Left pre-central gyrus	536	3.64	72	65	47
	Left post-central gyrus					
T-1	Left pre-central gyrus	1590	3.57	50	63	59
	Left post-central gyrus					
	Left juxtapositional lobule cortex (formerly supplementary motor cortex)					
	Left paracingulate gyrus					
	Left cingulate gyrus, anterior division					
	Left insular cortex	1468	3.8	68	58	49
	Left inferior frontal gyrus, pars opercularis					
	Left pre-central gyrus					
	Left temporal pole					
	Left superior temporal gyrus, anterior division					
	Left post-central gyrus					
	Left frontal operculum cortex					
	Left central opercular cortex					
	Left planum polare					
	Left insular cortex	1403	3.94	73	44	48
	Left superior temporal gyrus, posterior division					
	Left post-central gyrus					
	Left supramarginal gyrus, anterior division					
	Left supramarginal gyrus, posterior division					
	Left central opercular cortex					
	Left parietal operculum cortex					
	Left heschl's gyrus (includes H1 and H2)					
	Left planum temporale					
	Right pre-central gyrus	1346	3.92	21	49	45
	Right superior temporal gyrus, posterior division					
	Right post-central gyrus					
	Right supramarginal gyrus, anterior division					
	Right supramarginal gyrus, posterior division					
	Right central opercular cortex					
	Right parietal operculum cortex					
	Right planum temporale					
	Right lateral occipital cortex, superior division	700	3.4	25	19	38
	Right lateral occipital cortex, inferior division					
	Right occipital pole					
	Left lateral occipital cortex, superior division	516	3.6	64	22	43
	Left lateral occipital cortex, inferior division					
	Left occipital pole					
	Right putamen	449	3.39	31	62	28
	Right amygdala					
T0	Left insular cortex	5289	3.8	58	−6	20
	Left inferior frontal gyrus, pars opercularis					
	Left pre-central gyrus					
	Left temporal pole					
	Left post-central gyrus					
	Left supramarginal gyrus, anterior division					
	Left central opercular cortex					
	Left parietal operculum cortex					
	Left planum polare					
	Left planum temporale					
	Left thalamus					
	Brain-stem					
	Right thalamus					
	Right pallidum					
	Right insular cortex	2160	3.62	62	4	−4
	Right inferior frontal gyrus, pars opercularis					
	Right pre-central gyrus					
	Right temporal pole					
	Right superior temporal gyrus, anterior division					
	Right superior temporal gyrus, posterior division					
	Right post-central gyrus					
	Right supramarginal gyrus, anterior division					
	Right frontal operculum cortex					
	Right central opercular cortex					
	Right parietal operculum cortex					
	Right planum polare					
	Right heschl's gyrus (includes H1 and H2)					
	Right planum temporale					
	Left lingual gyrus	1443	4.01	22	−70	−18
	Left temporal occipital fusiform cortex					
	Left occipital fusiform gyrus					

Structures defined according to the Harvard-Oxford Cortical and Subcortical Structural atlases.

Using random regressors on our data, no significant activation could be found on the group level using both the cluster-corrected level of significance and the uncorrected voxel-level threshold of *Z* > 3 (data not shown).

### Functional data “RSN”

**Figure 4:**
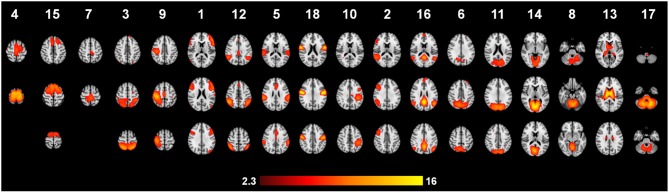
**Independent component maps with a clear neuroanatomical pattern**.

**Figure 5:**
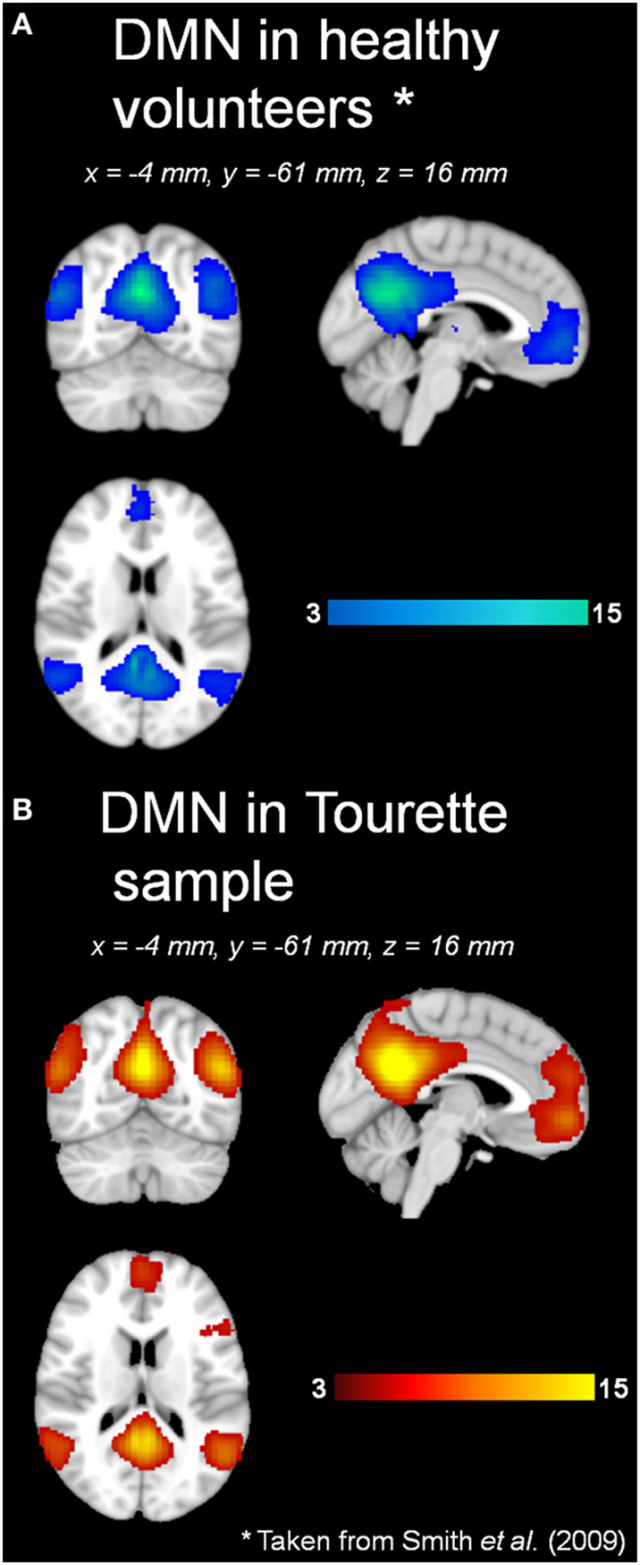
**DMN in (A) healthy volunteers and (B) Tourette patients**. ^*^Data taken from Smith et al., 2009.

**Figure 6:**
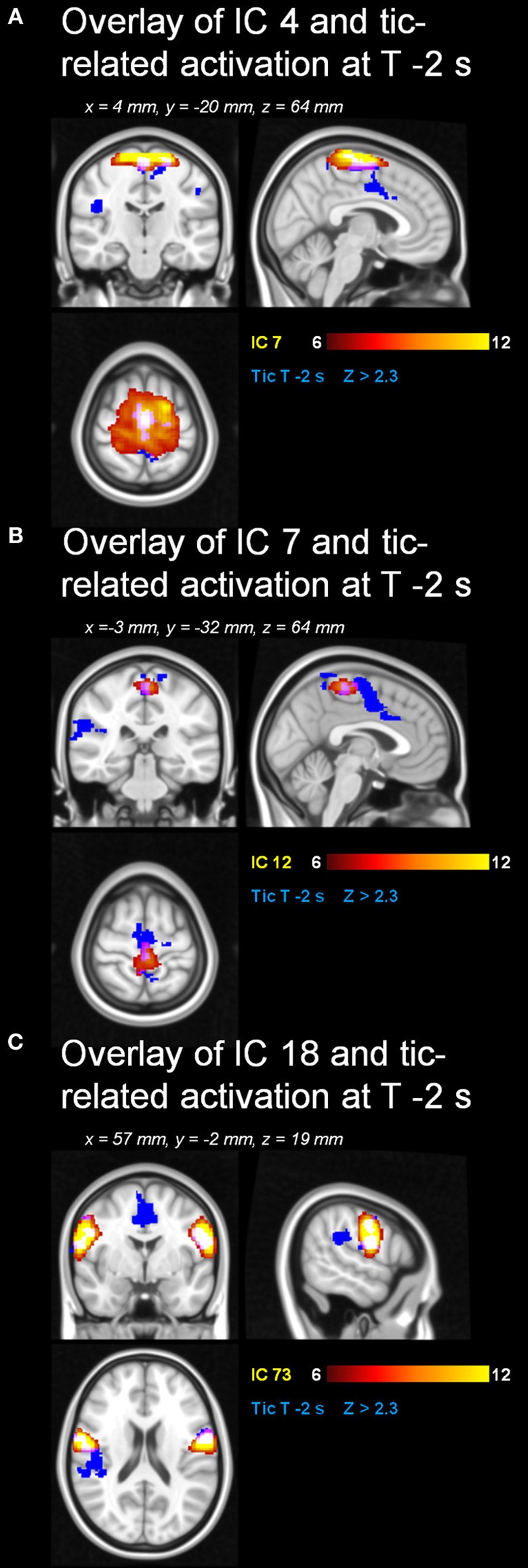
**Resting state networks with spatial overlap with tic-related activity**. Spatial overlap of the RSNs **(A)** 4, **(B)** 7 and **(C)** 18 with tic-related activity at “t-2”.

**Figure 7:**
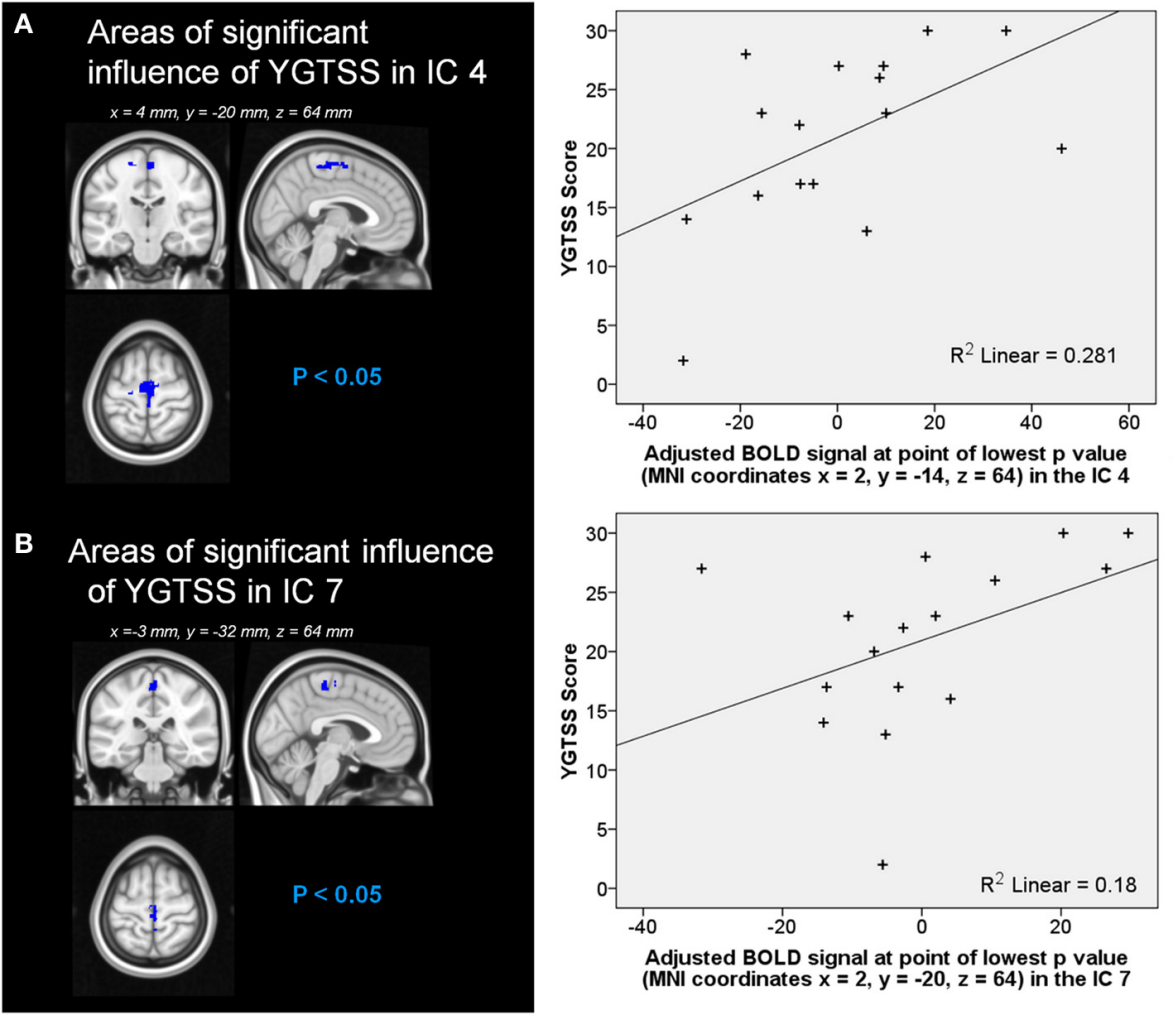
**Influence of YGTSS on network strength of RSNs **(A)** 4 and **(B)** 7**. Graphical representation of the YFTSS in function of **(A)** the adjusted BOLD signal at point of lowest *p-value* (MNI coordinates *x* = 2, *y* = −14, *z* = 64) in the RSN 4 and **(B)** the adjusted BOLD signal at point of lowest *p-value* (MNI coordinates *x* = 2, *y* = −20, *z* = 64) in the RSN 7.

Automatic dimensionality detection yielded a total of 74 components, 18 of which showed a clear neuroanatomical pattern encompassing neuroanatomical modules such as the primary visual cortex and the motor network (Figure 4), very well in keep with previous reports (Smith et al., 2009). The DMN was also clearly present (Figures 4, 5). The RSNs, maps no. 4, 7 and 18 showed spatial overlap with activation related to tic generation (contrast t-2, see below) (Figure 6). Dual regression analysis of these intersections revealed a significant influence of YGTSS motor + vocal on network strength in ROIs of RSNs 4 and 7, but not 18 (Figure 7), i.e., patients with higher YGTSS scores showed higher network scores of RSNs evoked by spontaneous fluctuations in those regions that become active 2 s prior to observable tic activity. The correlation coefficients between adjusted RSN/BOLD-signal and YGTSS were *r* = 0.53 (*p* = 0.031, FDR-corrected for multiple comparisons) for RSN 4 at coordinates *x* = 2, *y* = −14, *z* = 64 (MNI space) and *r* = 0.42 (*p* = 0.04, FDR-corrected) at coordinates *x* = 2, *y* = −20, *z* = 64 (MNI space) for RSN 7, respectively.

## Discussion

### Imaging the where and when of tic generation

#### Imaging pattern 2 s prior to tic onset—cortical origin of tic

The MR-compatible video system approach qualitatively revealed a temporo-spatial pattern of tics with cortical origin, following the long standing hypothesis of the cortico-striato-thalamo-cortical circuit. Two seconds before tic onset, neuronal activation starts in cortical areas, such as the SMA (BA6 mesial), ventral primary motor cortex (BA6 lateral), primary sensorimotor cortex (BA3+4) and parietal operculum. These findings are in line with a prior study by Bohlhalter and co-workers, in which a similar pattern is described 2 s before tics (Bohlhalter et al., 2006). In their fMRI study (1.5 T, 10 adult TS patients), the group also identified activity of the SMA, the pre-central and post-central gyrus, and the parietal operculum, among other structures. They discuss as pathophysiological mechanism the disinhibition of the motor system in TS, e.g., as assessed by transcranial magnetic stimulation (TMS) showing a deficient intracortical inhibition and shortened cortical silent period (Ziemann et al., 1997; Heise et al., 2010). The role of the SMA in the generation of tics was already highlighted in early ^18^FDG-PET studies. Braun and co-workers compared 16 adult TS patients with healthy controls. Increased metabolic activity was shown in the SMA, lateral premotor and Rolandic cortices (Braun et al., 1993). Although the temporal resolution of ^18^FDG-PET is poor, an elegant MRI-based approach by Hampson and co-workers complements the analysis of SMA contribution to tic generation (Hampson et al., 2009). After individual localizer scans of the primary motor cortex in TS patients, they analyzed in the framework of a correlational analysis the SMA activation time course in sliding time windows in comparison to tic onset. This approach revealed an increased functional interaction between M1 and SMA suggested by Hampson and co-workers as a pathophysiological signature of TS. This interaction was already present during movement preparation. The activation time course in the SMA indicated that this area exhibits an abnormally elevated activity in the seconds preceding and following tic execution (Hampson et al., 2009). These findings are in line with the activation patterns prior to a movement obtained by the means of magnetoencephalography (MEG) in the study of by Franzkowiak and co-workers in an adult TS sample (Franzkowiak et al., 2012).

Electrical stimulation of the SMA in humans is known to trigger motor responses as well as subjective-sensory responses such as an urge to perform a movement or anticipation that a movement was going to occur (Fried et al., 1991). Motor responses, elicited by electrical SMA stimulation, were classified by Fried and colleagues in three different categories: (a) simple, (b) regional, and (c) complex. Simple responses were defined as “discrete movements involving one joint or restricted to the digits of one extremity. Regional responses were classified as involving several joints but were confined to e.g., the face, neck, trunk or one upper/lower extremity. Motor responses including several body regions were classified as complex.” Electrical stimulation of the SMA in humans suffering from treatment resistant epilepsy resulted in 40 out of 129 stimulation points in simple motor or sensory responses, in 49 out of 129 in regional responses and in 28 out of 129 in complex responses. This detailed analysis shows that electrical stimulation or a proposed hyperactivity due to disinhibition of the SMA in TS patients is able to induce simple and complex tics, as well as sensory phenomena like the urge to tic. Moreover, the time delay between SMA activity and actual movement onset, as assessed through our MR-compatible video system, was previously described in the detailed and carefully controlled (i.e., for stimulus spread) study by Fried and co-workers (Fried et al., 1991). Eccles proposed already in 1982 a salient role of the SMA in programming and initiation of movement (Eccles, 1982). Early regional cerebral blood flow (CBF) studies using ^133^Xe discuss CBF changes in the SMA in association with programming of a sequence of movements without actually executing it (Roland et al., 1980).

Apart from the SMA, the origin of sensory urges, often reported by TS patients, might have a neuronal correlate in an early activation of the primary sensorimotor cortex in absence of an external stimulus. A spontaneous discharge due to disinhibition without external stimulation would comply with the general hypothesis of Donald Cohen, according to which TS is the result of a lack of inhibition, which has been proven by, e.g., electrophysiological studies applying TMS (Ziemann et al., 1997; Heise et al., 2010).

Another candidate for neuronal correlates of sensory phenomena prior to motor tics is the parietal operculum, which has been identified in our data and others, e.g., Bohlhalter and co-workers, as being active 2 s prior to a tic (Bohlhalter et al., 2006). The parietal operculum consists of different subregions. One is area S2, which is described as a somatosensory perceptive area that has strong connections with the inferior parietal cortex (Disbrow et al., 2003; Eickhoff et al., 2010). Electrical stimulation of the parietal operculum results in somatosensory experiences (Isnard et al., 2004; Eickhoff et al., 2010). The parietal ventral (PV) area is also mutually involved in sensory-motor integration and has denser connections with frontal motor and premotor cortices (Qi et al., 2002; Eickhoff et al., 2010); especially the latter function could be crucial for tic generation in TS.

#### Imaging pattern 1 s prior to tic onset—potential modification of tic outcome?

One second before the onset of tics, the anterior cingulate, the putamen, the insula, the amygdala, the cerebellum and the extrastriatal-visual cortex exhibited activation. For this 1-s time frame, there is no direct comparison in the literature available. The structures identified in this study that are active with tic onset were also reported in others, although with a lower time resolution or the use of different analysis approaches (Stern et al., 2000; Bohlhalter et al., 2006; Lerner et al., 2007). However, the activation patterns derived from this study correlate well with clinical reports of patients and observations made by family members and physicians of TS patients. In a single-case fMRI study, the ACC was activated during tic suppression (Kawohl et al., 2009a), similarly to a prior group fMRI study by Peterson and co-workers (Peterson et al., 1998).

Patients often report that they are in part able to suppress tics—for how long and to what degree is highly variable from individual to individual. Thus, the ACC activation in the tic cascade may indicate a failure of inhibitory tonus, and consequently, 1 s later tics occur as the video depicts. The ACC is known to play a pivotal role in motor functions as well as in emotion (Devinsky et al., 1995b). Electrophysiological studies have proven that the ACC regulates movement and is engaged in premotor functions (Luppino et al., 1991; Devinsky et al., 1995b). Furthermore, it is an essential structure for the transition from early premotor to behavioral states (for review please see Devinsky et al., 1995a). In addition to motor output regulation, it acts as “both an amplifier and filter, interconnecting the emotional and cognitive components of the mind” (Devinsky et al., 1995b). Having in mind the clinical characteristics of tic suppression and influence of affective and cognitive states on tic frequency the activation of the ACC 1 s prior to a tic fits well into the proposed underlying neuronal circuit. This is in line with results from Church et al. (2009) who describe the two different control systems that exist in TS and healthy volunteers: the frontoparietal and the cingulo-opercular. Both networks are compromised in TS, the frontoparietal to a larger degree than the cingulo-opercular. The dorsal ACC is one part of the cingulo-opercular system that shows disconnectivity in TS patients (Church et al., 2009).

The putamen is the first part of the basal ganglia involved in tic generation. Although structural alterations in different parts of the basal ganglia have been well replicated (Peterson et al., 2003; Plessen et al., 2009), in our imaging data, the basal ganglia do not seem to be involved in the early phases of tic generation. Given the positive outcome in a small case series of deep brain stimulation in treatment resistant cases, the implantation of electrodes seems to work within the cortico-striato-thalamo-cortical circuit (Neuner et al., 2009; Ackermans et al., 2011). Current research results suggest that it does not necessarily require intervention at the origin of tics.

In our data, the insula was also identified as a structure exhibiting activation 1 s prior to tic onset. The insula could, together with the SMA, be the neuronal substrate of premonitory urges. The phenomenon of premonitory urges has been compared in the literature to the phenomenon of itching (Bohlhalter et al., 2006) and neuroimaging studies identified a network comprised of the ACC, parietal operculum, thalamus and the insula as the origin of unpleasant sensations associated with itching and the urge to scratch, or pain (Hsieh et al., 1994; Kwan et al., 2000; Derbyshire et al., 2004; Bohlhalter et al., 2006). The insula is thought to be functionally responsible for “behaviors which require an integration between extrapersonal stimuli and internal milieu” (Mesulam and Mufson, 1982a; Lerner et al., 2007). This hypothesis was confirmed in clinical settings where tight interaction between tics' frequency and severity, emotional state of the patient and actual situation (sitting in a lecture hall, driving a car) can be observed. The insula is part of widespread networks and is tightly connected with cortical and subcortical areas. It has also reciprocal connections with primary, association, premotor and paralimbic cortices, and has multiple connections with subcortical structures including amygdala, claustrum, and thalamic nuclei (Mesulam and Mufson, 1982a,b; Mufson and Mesulam, 1982; Craig, 2002, 2009). The insula exhibits tight connections to the amygdala, which also showed significant activation 1 s prior to a tic. The emotional input for the insula's integration between extrapersonal stimuli and internal milieu might be delivered via the amygdala. The activation of the amygdala is an interesting finding as well, since both clinical and neuroimaging studies support its involvement in tic generation. Patients often report that situations of emotional distress, but also of great joy, can dramatically increase the frequency and intensity of tics. This might be modulated by amygdala activation prior to a tic. This assumption is also supported by neuroimaging data. Werner and colleagues reported altered functional connectivity of the amygdala in TS patients: in a task related fMRI design, TS patients showed a striking hypersensitivity of the amygdala in response to facial emotional expressions (Neuner et al., 2010a; Werner et al., 2010). In the studies of Wang and Lerner (Wang et al., 2011; Lerner et al., 2012) the amygdala is also discussed as a mutual part in tic generation (Vandewalle et al., 1999; Ackermans et al., 2011, 2013a,b). Thus, in summary, the ACC, the insula and the amygdala may be regarded as potentially modifying relay points in tic generation.

#### Imaging pattern at tic onset—output via thalamic nuclei?

With tic-onset, the thalamus, primary motor and somatosensory cortices, and the central operculum exhibited activation. This is in line with ^15^O-PET results by Stern, and Lerner and co-workers (Stern et al., 2000; Lerner et al., 2007) and MR-based investigations by Hampson and co-workers (Hampson et al., 2009). A recent study applying ^11^C-flumazenil PET in TS individuals described the following pattern for tic generation: decreased binding bilaterally in the ventral striatum, globus pallidus, thalamus, amygdala, and right insula; increased binding in the substantia nigra, periaqueductal gray, posterior cingulate cortex and cerebellum. This PET study broadens the scope beyond the role of the dopaminergic system into the GABAergic transmitter system (Felling and Singer, 2011), the master of inhibition. The decreased pattern in GABA_A_ binding potentials includes the subcortical components of the cortico-striato-thalamo-cortical circuit including the amygdala and insula for emotional and somatosensory integration in an impressive fashion. Our results are also matched by the success of the still experimental treatment approach of deep brain stimulation in TS patients. Different target points in the thalamic nuclei, the globus pallidus internus, and nucleus accumbens (Vandewalle et al., 1999; Neuner et al., 2009; Ackermans et al., 2011, 2013a,b) exist, and tic reduction was reported to be as high as 80%, as assessed by the YGTTS (Leckman et al., 1989).

### Default-mode network and premotor RSNs

Our resting state analysis approach revealed the presence of the classic DMN, and other typical pre-described RSNs in adult TS patients. To the best of our knowledge, these results are the first with regard to RSNs in adult TS patients. Temporal concatenation ICA proves to be a valuable tool in this clinical setting, too, as it is very robust against influence of noise in the data and enabled us to detect well over 20 neuroanatomically meaningful maps of functional connectivity. Visual inspection, and statistical comparison with a previously published set of RSNs, revealed the presence of the DMN in TS patients under resting conditions in almost identical form and extent as in the 36 subjects in which the canonical RSNs were derived from (Smith et al., 2009). This indicates a relative preservation of the DMN—an important finding given the fact that the DMN is disrupted in a variety of neurological conditions such as Alzheimer's (Li et al., 2002; Greicius et al., 2004) or Parkinson's disease (Van Eimeren et al., 2009; Delaveau et al., 2010).

The pure striatothalamic and thalamic RSNs identified in our TS patients are a core element of the cortico-striato-thalamo-cortical circuit. On a descriptive level, the identification of these subcortical components complement the study by Church and co-workers (Church et al., 2009), in which predefined cortical regions of interest exhibited immature and anomalous patterns of functional connectivity. This RSN certainly deserves more attention in further studies.

Our parametric RSN analysis of motor-area ICAs complements the previously discussed role of the SMA in the generation of tics very nicely. First, we identified three ICAs that showed substantial overlap with pre-tic activation patterns. Of these, only two showed a significant correlation with YGTSS scores, and within these, those correlations were restricted to the mesial aspect of BA6, e.g., the SMA. Put differently, it seems that within SMA, RSN connectivity seems to be altered in a disease severity dependent fashion, and this alteration might contribute to the generation of tics in those areas.

Given that the resting state activity might use up well over 90% of the energy budget of the brain, the PET results by Braun et al. (1993) highlighting metabolic alterations of the SMA could well mirror the altered resting state activity in SMA characterized in our study.

It has been shown previously that ongoing activity, as represented by RSNs, influences both evoked activation and behavior (for a review see Sadaghiani et al., 2010). Esposito and colleagues reported in Parkinson patients a positive relationship between neuronal activity in the sensorimotor network, especially in the SMA and clinical motor function (Esposito et al., 2013). Thus, we hypothesize that in TS aberrant RSN activity could contribute to a larger propensity of SMA to generate tic-related activity. As there was no significant correlation between total tic-time (contributing to “evoked” neuronal signal in SMA) and YGTSS, we argue that this result is not an artifact from the tics themselves, but representative of a disease-specific property of SMA. Therefore, it would be interesting to see the effect of different therapeutic approaches to TS on RSN integrity measures, particularly in SMA, and their relation to resulting tic activity and disease severity scores.

### Limitations of the study

Since we aimed to investigate the neuronal correlates of tics, this study included moderate to severely affected TS patients in order to gain statistical significant number of events i.e., tics for the analysis of data. However, since tics result in motion artifacts in the fMRI data, a substantial number of patients had to be excluded. We also introduced objectivity in the definition of onset and duration of tics by using an MR-compatible camera system, and by including this information as regressors in the analysis of data. Nevertheless, the use of the camera system also introduced a potential factor contributing to data loss. Furthermore, by including moderate to severely affected TS individuals, the sample exhibited a high ratio of medicated individuals. The medication itself falls into different substance classes with different half lives, metabolism, etc. We therefore decided against the introduction of 4 additional covariants of no interest in the GLM and resting state analysis. However, despite the medication and differences in percentage of medication in the GLM sample and resting state sample we were able to describe robust neuronal activation for the where and when of tic generation as well as for the RSNs.

Even after optimizing the detection of onset and duration of tics via the MR-compatible video camera system, the low temporal resolution of any fMRI-based approach has to be acknowledged. The low TR of 3.200 ms is somewhat mitigated by the oversampling of the data induced by the natural jitter of the events of interest (e.g., tics), however. While the course of the activation patters shows a nice flow along previously described circuits, is of clear neuroanatomical plausibility (e.g., premotor areas activating before primary sensorimotor areas) and is a replication of previous work, it has to be acknowledged that the time-bins of 1 s each are both arbitrary and not in the actual range of neuronal signaling. However, peristimulus plots (see Supplementary Figure 2 for an example) show the typical time-course of an HRF around each of the time-points chosen (noise notwithstanding), making artifactual results (e.g., by slice-timing artifacts or hugely different shapes of the HRF in different locations of the brain) unlikely. The fact that all results are valid on the second level (corrected for multiple comparisons) indicates that our results hold true for the entire cohort, making spurious results even further unlikely. This is further corroborated by our introduction of completely random events in an additional analysis, which did not show any significant results (corrected and uncorrected) on the second level.

## Conclusions

Using an fMRI approach controlled by video-camera, we demonstrate that the temporal pattern of tic generation follows the cortico-striato-thalamo-cortical circuit. In our data, cortical structures precede subcortical activation. Network integrity in two premotor RSNs overlapping with tic preparatory activation patterns exhibited a significant correlation with YGTSS scores in SMA, but not elsewhere, indicating an interaction between ongoing brain activity, tic-related activity and clinical disease measures in that particular area. Further researches, particularly in longitudinal prospective studies, as well as in designs employing therapeutic interventions are necessary to determine how alterations of RSNs in TS patients might function as state or trait markers of the disorder.

## Financial disclosure

Irene Neuner, Cornelius J. Werner, Jorge Arrubla, Tony Stöcker, Corinna Ehlen and Hans P. Wegener report no conflict of interest. N. Jon Shah reports funding from the BMBF and Siemens for the 9.4T MR/PET (magnetic resonance/positron emission tomography) project. Frank Schneider received compensation as a consultant for Janssen-Cilag, AstraZeneca, and Otsouka, manufacturers of antipsychotic medication. Frank Schneider received compensation for scientific talks or contribution in a prize jury by Janssen-Cilag, Wyeth, and AstraZeneca. Frank Schneider received funding for investigator initiated projects from AstraZeneca, Lilly and Pfizer.

### Conflict of interest statement

The authors declare that the research was conducted in the absence of any commercial or financial relationships that could be construed as a potential conflict of interest.

## Supplementary material

Supplementary Figure 1 **Framework of the study: event related design, tics occur at random (so onsets are well jittered), on average number of events n = 39.5, MR-compatible camera system recording**.

1) Start with identifying tic onsets from the recorded video acquired simultaneously with imaging.

2) This serves as a time stamp on the data.

3) Data are aligned according to time stamp.

4) Data are sorted according to increasing jitter.

5) This insures data can be viewed along the negative time axis and the positive time axis, taking the origin of the tic to be 0 of time.

6) Any pertinent inaccuracies in the positive time direction are the same as in the negative time direction and importantly these are the same at t0, t-1, and t-2. There are no systematic differences.

Graphics: **(A)** Timeline of hypothetical tics 1, 40 and “n” with data-points marked. **(B)** Timelines aligned around the tic occurrence. **(C)** Integration of the 3 timelines and their contribution to the HRF of *T* = 0 s, *T* = −1 s, and *T* = −2 s. Despite having a TR of 3.2 s, it is possible to delineate neuronal events that are separated by only a time increment of 1 s due to the oversampling of the HRF over the course of the experiment which results from the natural “jitter” of the tic events. This jitter leads to a near-complete sampling of the HRF at each time-point.

Supplementary Figure 2 **BOLD signal time-course in subject 6 for. (A)** t-2, **(B)** t-1, and **(C)** tic onset. The time-course was extracted at the voxel with the highest z-score, i.e., the best fit with the model showing that HRFs are comparable even in different locations.
