# Supplementary material for: Imaging the where and when of tic generation and resting state networks in adult Tourette patients
Source: Front Hum Neurosci. 2014 May 28;8:362. doi: 10.3389/fnhum.2014.00362 (PMC4035756; doi:10.3389/fnhum.2014.00362)
Supplement: Supplementary Figure 1 — Framework of the study: event related design, tics occur at random (so onsets are well jittered), on average number of events n = 39.5, MR-compatible camera system recording. [file Presentation1.ZIP › 74803_Neuner_table 1.DOCX]

**Supplementary Table 1: Neuronal networks underlying tic generation in Tourette studies**

| **Literature** | **fMRI / PET** | **Design** | **Sample description** | **Main findings** |
| --- | --- | --- | --- | --- |
| Peterson et al. 1998 | fMRI | Instruction to inhibit tics and let tics go freely in 40 seconds blocks; no monitoring of performance | N=22 adult TS (11f, 11m, aged 35.7±10.9 years), 15 medication free, 10 OCD, 3 ADHD as child not as adult | Contrast non-suppressed vs. suppressed showed a reduction of  signal intensity in the basal ganglia and thalamus and  increased activation in the midfrontal, middle and superior temporal  gyrus, anterior cingulate cortex and inferior occipital cortices |
| Bohlhalter et al. 2006 | fMRI | Instruction to let tics occur freely;  analysis of tics 2 seconds prior to tics and at tic occurrence | N=10 adult TS (6f, 4m, aged 31±11 years), 9 medication free, 4 OCD, 2 ADHD | 2 seconds before tic occurrence: SMA, parietal operculum,  insular cortex, anterior cingulate cortex activated  at tic onset: sensorimotor areas including superior parietal lobule bilaterally, dorsolateral prefrontal cortex, parietal operculum, SMA,  insula, putamen, vermis and substantia nigra |
| Hampson et al. 2009 | fMRI | Instructions to allow tics to occur freely; ROI based approach SMA; correlational analysis of co-activated voxels with SMA during tics | N=16 adult TS/CTD (3f, 13m, aged 30±9.7 years), 8 medication free, 2 OCD, 2 ADHD and major depressive disorder vs. 10 healthy controls | Increased functional interaction between M1 and SMA as a pathophysiological marker for TS; alterations present during  preparation and execution of voluntary movements indicating  increased motor-cortical interaction in TS |
| Kawohl et al. 2009 | fMRI | Tic suppression | 1 male, 28 years, no medication, comorbidities NS | Anterior cingulate cortex activated during tic suppression |
| Church et al. 2009 | fMRI | Graph theory approach;  basal ganglia not included as a hub | N=33 children TS (8f, 25m, aged 12.7 ±0.76), 22 on medication, 17 with comorbidities  vs. 42 healthy controls | Two control networks: fronto-parietal network governing rapid  adaptive online control, cingulo-opercular network governing set-maintenance; adolescents with TS have immature pattern,  particularly in the frontoparietal network, aberrant connections |
| Mazzone et al. 2010 | fMRI | Inhibition and disinibition of “semi”- involuntary eyeblinks, block design, 40 seconds; member of study team observes blink performance via mirror in the MR suite | N= 22 children TS (3f, 19m, aged 13.1±2.6, 1 medication free, 21 OCD, 11 ADHD, 4 OCD/ADHD) vs. 21 matched healthy controls,  N= 29 adult TS (12f, 17m, aged 35.1±11.1, medication free, OCD, ADHD, OCD/ADHD NS vs. 48 matched healthy controls | During blink inhibition TS individuals showed stronger activation in  the frontal cortex and the striatum. Age effect: activation level in the dorsolateral and inferolateral prefrontal cortex and caudate nucleus increased with increasing age |
| Wang et al. 2012 | fMRI | Spontaneous vs. imitated tics  ICA analysis, Granger causality | N=13 adult TS (11f, 11m, aged 33.5±13.3 years), 7 medication free, 6 OCD, 2 ADHD, vs. 21 matched healthy control subjects | Stronger pattern of activation in TS patients in the sensorimotor  cortex, putamen, pallidum and substantia nigra, positive correlation  with tic severity;  Comparison “original tics” vs. imitated tics: stronger activation in  posterior parietal cortices |
| Braun et al. 1993 | ^18^FDG-PET | Tics occur freely | N=16 adult TS (2f, 14m, aged 33±7 years), medication free, comorbidities NA vs. 16 healthy controls | Decreased metabolic activity in paralimbic and prefrontal cortices,  nucleus accumbens, ventromedial caudate, midbrain  Increased metabolic activity in SMA, lateral premotor, Rolandic  cortices |
| Braun et al. 1995 | ^18^FDG-PET | Tics occur freely | N=18 adult TS (2f, 16m, aged 33±7 years), medication free, comorbidities assessed | Complex behavioural symptoms such as OCD, impulsivity, coprolalia,  self-inflictious behavior correlate with increased metabolic activity in  the orbitofrontal cortices |
| Eidelberg et al. 1997 | ^18^FDG-PET | No specific instruction described | N=10 adult TS (5f/5m), 41.5±12.7 years, medication free, comorbidities NA vs. 10 healthy controls | Increased activity in lateral premotor, SMA and midbrain  Decreased activity in caudate nucleus, thalamus, putamen, globus pallidus and hippocampus |
| Jeffries et al. 2002 | ^18^FDG-PET | Connectivity analysis | N=18 adult TS (2f/16m, 33±7 years), medication free, 11OCD, ADHD NA vs. 16 healthy controls | Altered patterns of connectivity for the ventral striatum, primary motor areas, somatosensory association areas and the insula |
| Stern et al. 2000 | ^15^O-PET | Tics occur freely, video controlled approach | N=6 adult TS (0f,6m, aged 36.7±10.9 years), 4 medicated, including comorbi-dities | Activated structures included: medial and lateral premotor cortices, primary motor cortex, dorsolateral prefrontal cortex, inferior parietal  cortex, superior temporal gyrus, Broca-Area, anterior cingulate cortex, putamen, thalamus, insula, claustrum |
| Lerner et al. 2007 | ^15^O-PET | Tics occur freely | N=9 adult TS (2f, 7m, aged 20-44 years), medication free, 7 OCD, 5 ADHD, 1 obsessive compulsive personality disorder vs. 9 matched control healthy subjects | Activity in the cerebellum, thalamus, insula, putamen, caudate, pre-  and postcentral gyrus, SMA, anterior cingulate cortex |

Abbreviations: OCD= obsessive compulsive disorder; ADHD= attention deficit hyperactivity disorder; PET= positron emission tomography; fMRI= functional magnetic resonance imaging; FDG= Fluorodeoxyglucose, SMA= supplementary motor area, NA= not assessed, NS = not specified, CTD= chronic tic disorder
