# Supplementary figures and images for: Imaging the where and when of tic generation and resting state networks in adult Tourette patients
Source: Front Hum Neurosci. 2014 May 28;8:362. doi: 10.3389/fnhum.2014.00362 (PMC4035756; doi:10.3389/fnhum.2014.00362)

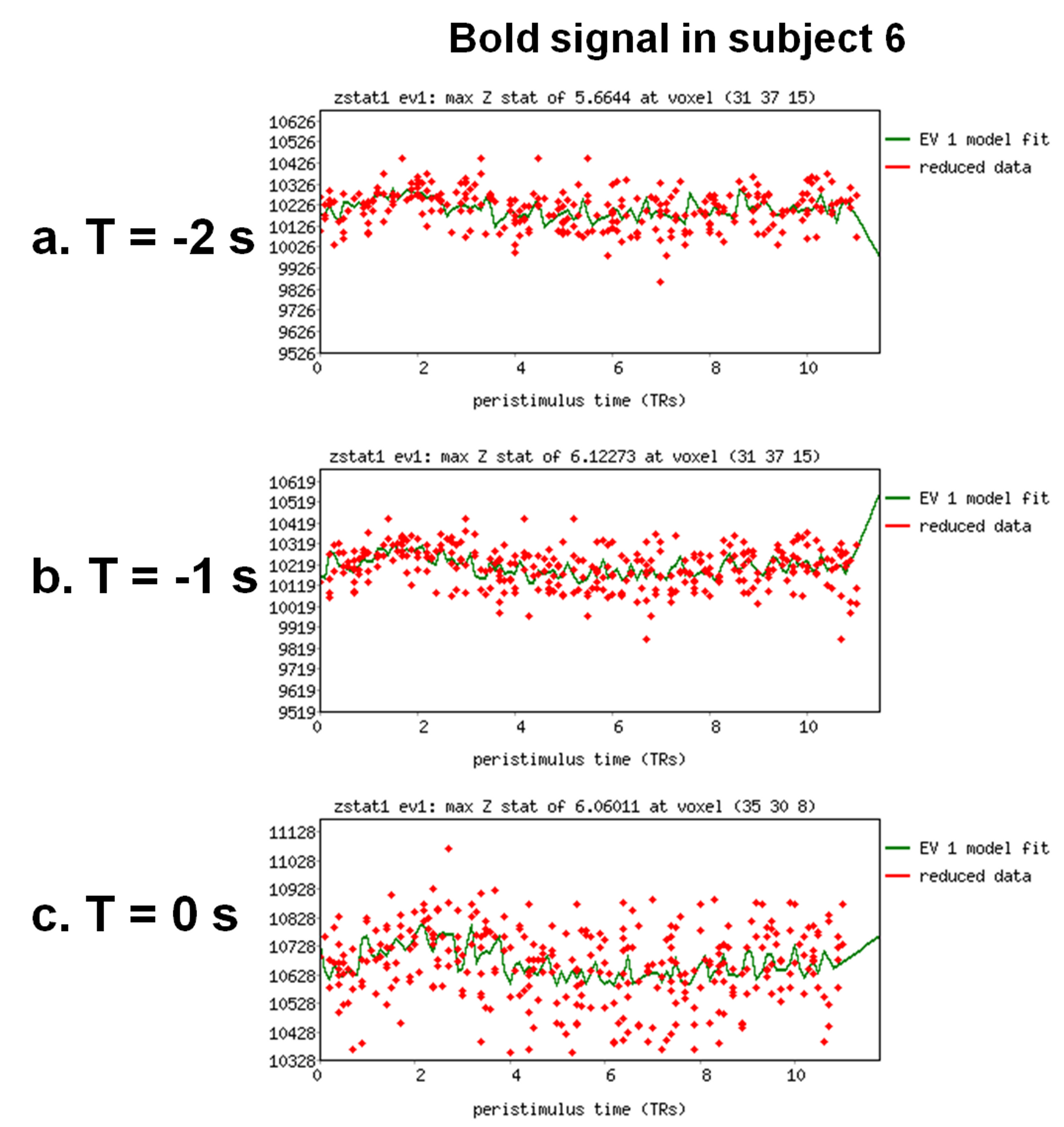

Supplement: Supplementary Figure 1 — Framework of the study: event related design, tics occur at random (so onsets are well jittered), on average number of events n = 39.5, MR-compatible camera system recording. [file Presentation1.ZIP › 74803_Neuner_Sup Fig 2.TIFF]
